# High histone crotonylation modification in bovine fibroblasts promotes cell proliferation and the developmental efficiency of preimplantation nuclear transfer embryos

**DOI:** 10.1038/s41598-024-61148-6

**Published:** 2024-05-04

**Authors:** Xiaoyu Zhao, Mengxin Du, Shanshan Wu, Zhiwen Du, Shuqin Liu, Lei Yang, Haoran Ma, Liguo Zhang, Lishuang Song, Chunling Bai, Guanghua Su, Guangpeng Li

**Affiliations:** 1 State Key Laboratory of Reproductive Regulation and Breeding of Grassland Livestock (R2BGL), Inner Mongolia University, 24 Zhaojun Rd., Hohhot, 010070 China; 2 College of Life Sciences, Inner Mongolia University, 24 Zhaojun Rd., Hohhot, 010070 China; 3 Agricultural Genomics Institute at Shenzhen, Chinese Academy of Agricultural Sciences, Shenzhen, 518000 China; 4 Institute of Animal Science, Chinese Academy of Agricultural Sciences, Beijing, 100193 China; 5 Ulanqab Agriculture and Animal Husbandry Bureau, Ulanqab Animal Husbandry Workstation, Ulanqab, 012000 Inner Mongolia China

**Keywords:** Cell biology, Cell death, Cell division, Cell growth, Post-translational modifications

## Abstract

Lysine crotonylation (Kcr) is a recently discovered histone acylation modification that is closely associated with gene expression, cell proliferation, and the maintenance of stem cell pluripotency and indicates the transcriptional activity of genes and the regulation of various biological processes. During cell culture, the introduction of exogenous croconic acid disodium salt (Nacr) has been shown to modulate intracellular Kcr levels. Although research on Kcr has increased, its role in cell growth and proliferation and its potential regulatory mechanisms remain unclear compared to those of histone methylation and acetylation. Our investigation demonstrated that the addition of 5 mM Nacr to cultured bovine fibroblasts increased the expression of genes associated with Kcr modification, ultimately promoting cell growth and stimulating cell proliferation. Somatic cell nuclear transfer of donor cells cultured in 5 mM Nacr resulted in 38.1% blastocyst development, which was significantly greater than that in the control group (25.2%). This research is important for elucidating the crotonylation modification mechanism in fibroblast proliferation to promote the efficacy of somatic cell nuclear transfer.

## Introduction

As one of the major disciplines in contemporary biology, epigenetic modification is widely believed to explain biological phenomena that classical genetics cannot explain. This mechanism operates independently of DNA sequence information, selectively executing DNA instructions based on epigenetic information to regulate heritable changes in biological phenotypes and modulate gene expression^1,2^. Histones, identified as basic proteins by the German scientist Kossel in 1884, were subsequently found to be susceptible to modifications such as lysine acetylation (Kac) by the discovery of the acetyltransferase Gcn5 in 1964^3,4^. Ongoing research has revealed a spectrum of modifications, including lactation, phosphorylation, methylation, propionylation, butyrylation, succinylation, malonylation, glutarylation, and crotonylation^4–10^. Histone modifications are predominantly located in euchromatin and heterochromatin regions and play a fundamental role in influencing crucial life processes, such as gene expression, cell proliferation, metabolism and disease^11–13^. Lysine crotonylation (Kcr), identified in 2011 by Prof. Yingming Zhao’s team, serves as a conserved chromatin marker associated with chromatin activation and silencing^14^. This modification is implicated in diverse physiopathological processes, including gene expression, DNA damage repair, spermatogenesis, and embryonic stem cell stemness^15,16^. Kcr-modifying enzymes, categorized as writers, erasers and readers, act on specific lysine residues, such as H3K4, H3K9, H3K18, H3K23, H3K27, H4K5, H4K8, H4K12 and H4K16^17–22^.

Kcr is one of the first nonacetyl histone lysine acylation modifications discovered that uses crotonyl-CoA as a substrate. Crotonyl-CoA is synthesized in the mitochondria or cytoplasm and serves as an intermediate metabolite in fatty acid oxidation and lysine or tryptophan metabolism^16,23^. Similarly, Simithy et al. reported that changes in the concentration of Cr-CoA affect Kcr levels in cocultured cells through enzymatic and nonenzymatic mechanisms^24^. Studies have demonstrated that the exogenous addition of croconic acid disodium salt (Nacr) during cell culture elevates Kcr modification by increasing the intracellular Cr-CoA content^16^. This modulation may affect various cellular processes, including embryonic differentiation, cell growth, proliferation and spermatogenesis^15,25–27^. In addition, knockdown of the crotonyl coenzyme A-producing enzyme acyl coenzyme A synthase short-chain family member 2 (*ACSS2*) in cultured cells significantly altered intracellular Kcr modification levels^28^.

Somatic cell nuclear transfer (SCNT) is an asexual reproduction technique with broad applications, from breeding to clonal therapy. Despite notable successes, the efficacy of SCNT remains low^29–32^. Fibroblasts from different sources are often used as donor cells during SCNT in domestic animals such as cattle and sheep^33^. Kim et al. demonstrated that the ageing and apoptosis-related characteristics of donor cells during culture affect the developmental efficiency of porcine SCNT embryos^34^. Therefore, we investigated whether the proliferative activity of donor fibroblasts can similarly affect the developmental efficiency of bovine SCNT embryos. Moreover, although previous studies have discussed how Nacr can regulate cell proliferation via Cr-CoA levels, the specific mechanism of Kcr modification and its impact on cell growth have not been fully explored.

Therefore, in the present study, we focused on the effects of adding Nacr to culture media on the viability of bovine donor fibroblasts and the development of SCNT embryos. The addition of exogenous Nacr to the culture medium may affect Kcr modification through the levels of Cr-CoA, increasing the viability and proliferation of bovine donor fibroblasts and subsequently increasing the efficacy of bovine SCNT embryo development. Our results demonstrated that the addition of Nacr during cell culture promoted Kcr modification, thereby increasing cell viability and proliferation and significantly increasing the blastocyst rate of bovine SCNT embryos. These findings help elucidate the mechanisms by which Kcr modification affects cell proliferation and provide new insights into increasing the efficacy of preimplantation development of SCNT embryos.

## Results

### Impact of Nacr on bovine fibroblast cell growth and viability

**Figure 1:**
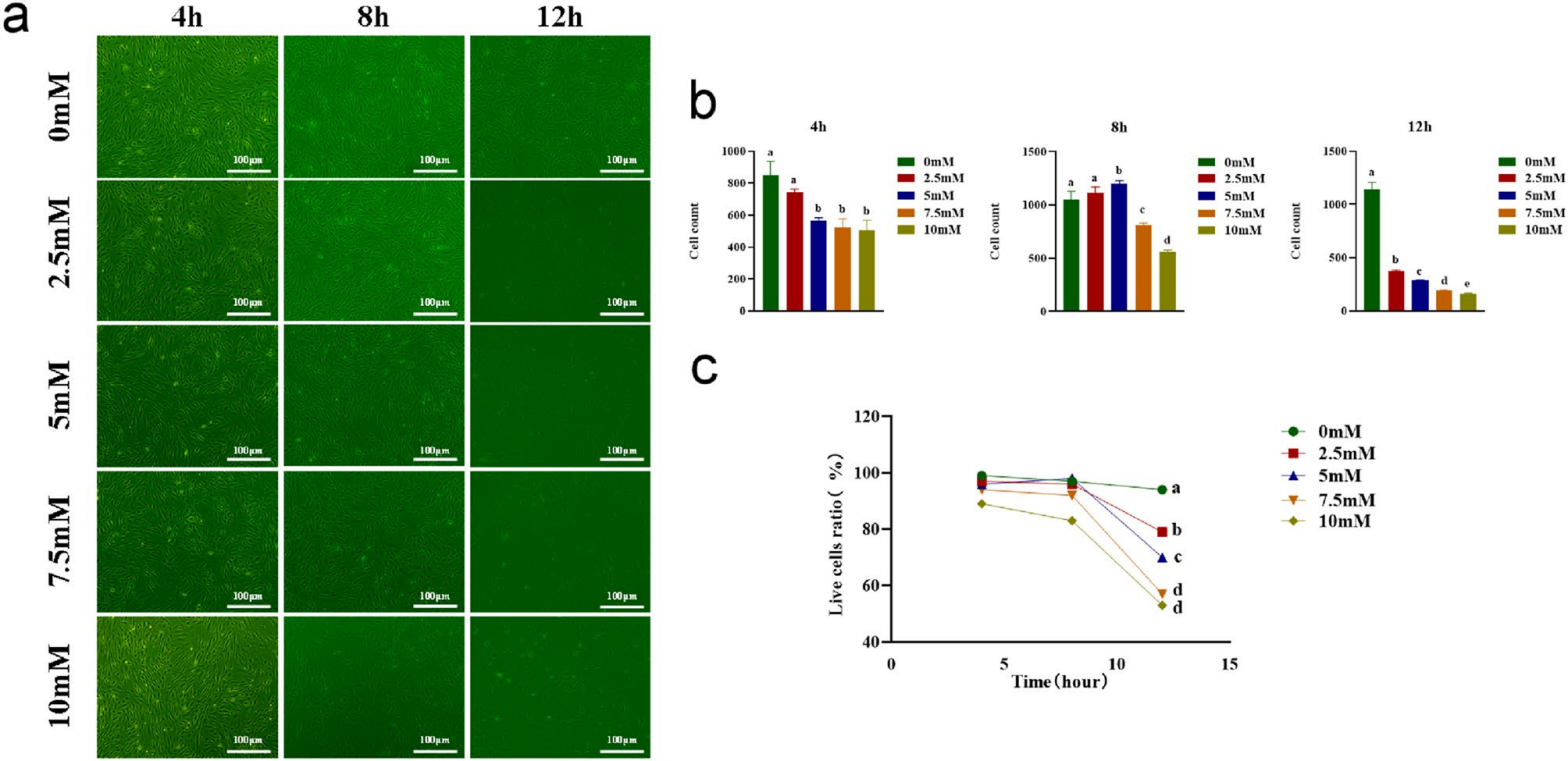
Effect of Nacr addition on bovine fibroblasts. (**a**) Morphology of bovine fibroblasts treated with Nacr at different concentrations and times; (**b**) Cell density analysis after the addition of various concentrations of Nacr for 4 h, 8 h, and 12 h; (**c**) Percentage of live cells after Nacr treatment at different concentrations and times. Letters with no significant differences were denoted by the same letter (*P* > 0.05), and different letters indicated significant (*P* < 0.05).

To evaluate the effects of exogenous Nacr on the density growth status of bovine fibroblasts, we conducted cell growth experiments. Bovine fibroblasts were exposed to 2.5 mM, 5 mM, 7.5 mM, and 10 mM Nacr for 4 h, 8 h, and 12 h in growth media. We investigated the possible changes in cell density during a specified time period. As depicted in Figure 1a,b, after 4 h of treatment, 5 mM, 7.5 mM, and 10 mM Nacr significantly reduced cell density compared to 0 mM. After 8 h of treatment, 2.5 mM Nacr had no significant effect on cell density, while significantly decreased in the 7.5 mM and 10 mM Nacr groups. However, when treated with 5 mM Nacr for 8 h, cell density significantly increased compared to 0 mM. After 12 h of treatment, compared to 0 mM Nacr, all Nacr concentrations (2.5 mM, 5 mM, 7.5 mM, and 10 mM) significantly reduced cell density. Statistical analysis of cell viability using trypan blue staining showed that after 4 h of culture, the group treated with 10 mM Nacr had a significantly lower number of viable cells. After 8 h of culture, the number of viable cells in the 5 mM Nacr group increased to the level of the 0 mM group, while the addition of 7.5 mM and 10 mM Nacr reduced the proportion of viable cells, and the proportion of viable cells in the 2.5 mM Nacr group did not change significantly. After 12 h of culture, compared to 0 mM, all Nacr concentrations (2.5 mM, 5 mM, 7.5 mM, and 10 mM) reduced the proportion of viable cells (Figure 1c).

### Impact of Nacr on bovine fibroblast proliferation and gene expression

**Figure 2:**
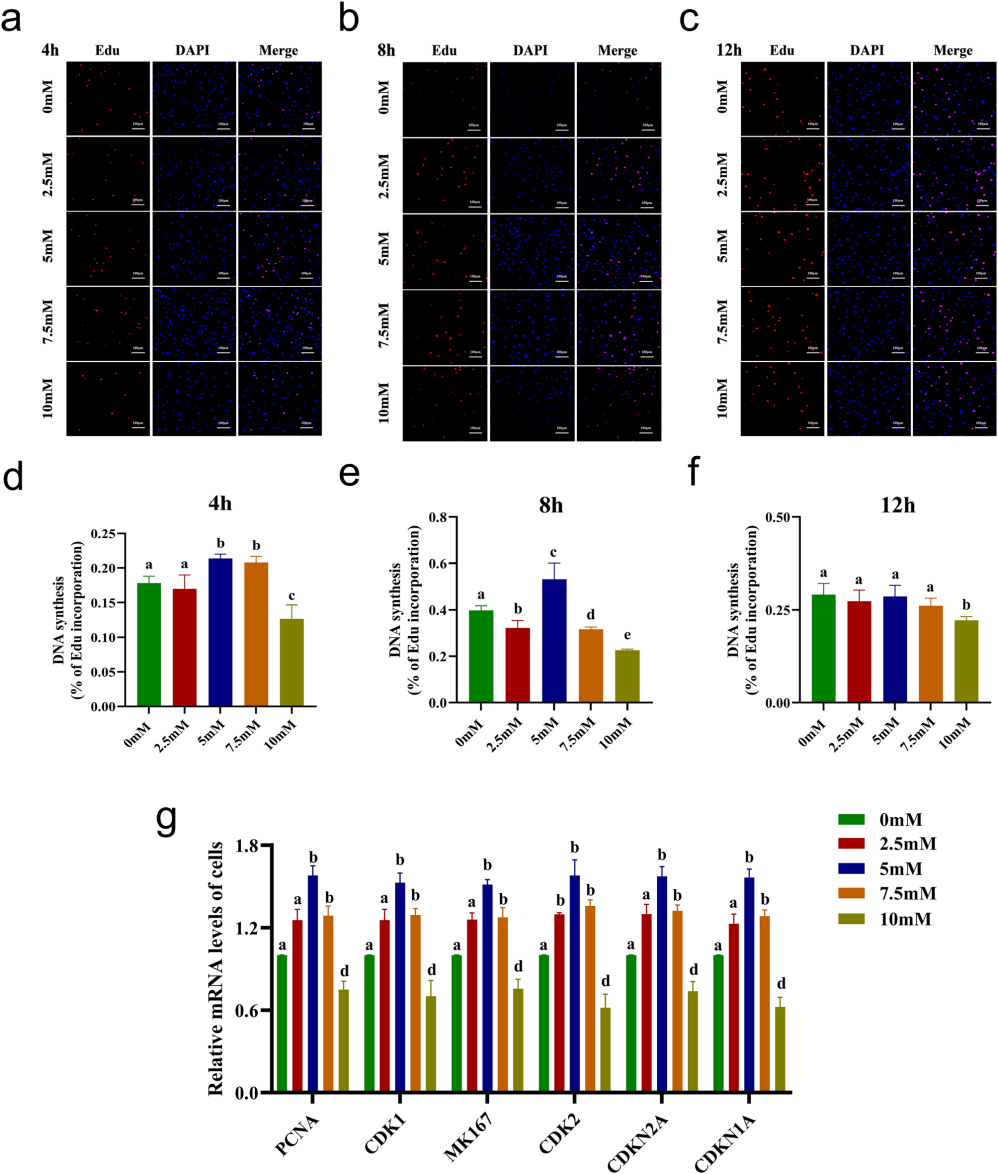
Proliferative capacity of bovine fibroblasts treated with Nacr. (**a**) Proliferative capacity of bovine fibroblasts detected by Edu after 4 h of Nacr at different concentrations; (**b**) Proliferative capacity of bovine fibroblasts detected by Edu after 8 h of Nacr at different concentrations s; (**c**) Proliferative capacity of bovine fibroblasts detected by Edu after 12 h of Nacr at different concentrations s; (**d**) Percentage of cells in DNA replication period after 4 h treatment with Nacr at different concentrations; (**e**) Percentage of cells in DNA replication period after 8 h treatment with Nacr at different concentrations; (**f**) Percentage of cells in the DNA replication phase after 12 h treatment with Nacr at different concentrations; (**g**) Proliferation gene expression in bovine fibroblasts after 8 h treatment with Nacr at different concentrations. Different letters represent significant differences.

As the alterations in cell morphology became evident with higher concentrations of Nacr and extended treatment periods, we postulated that these changes might influence the proliferative capacity of bovine fibroblasts. Therefore, we evaluated the proliferative capacity of fibroblasts treated with 2.5 mM, 5 mM, 7.5 mM and 10 mM Nacr for 4 h, 8 h and 12 h using EdU labelling. The results showed that 2.5 mM and 7.5 mM Nacr had no significant effect on cell proliferation after 4 h or 8 h of treatment. Notably, the addition of 5 mM Nacr increased the proliferation rate (Figure 2a,b,d,e). However, after 12 h of treatment, only 10 mM Nacr led to a decrease in cell proliferation, while 2.5 mM, 5 mM and 7.5 mM Nacr had no discernible effect, which may indicate that increased Nacr concentrations are cytotoxic to cell proliferation (Figure 2c,f).

Based on the above results, we found that different concentrations of Nacr had the most significant effect on cell proliferation after culturing the cells for 8 h. To determine the molecular mechanism underlying this effect, we treated fibroblasts with Nacr for 8 h, after which the expression of key proliferation-related genes was examined. These genes include proliferating cell nuclear antigen (*PCNA*), cyclin-dependent kinases (*CDK*)*1-2*, marker of proliferation Ki67 (*MK167*), and cyclin-dependent kinase inhibitor (*CDKN*)*1-2A*. Figure 2g shows that 2.5 mM, 5 mM and 7.5 mM Nacr significantly upregulated the mRNA expression of *PCNA*, *CDK1*, *MK167*, and *CDK2* (*P *< 0.05) while suppressing the expression of *CDKN1A* and *CDKN2A* (*P *< 0.05). Notably, the effect of 5 mM Nacr was the most significant (*P *< 0.01). In contrast, 10 mM Nacr decreased the expression of *PCNA*, *CDK1*, *MK167*, and *CDK2* and promoted the expression of *CDKN1A* and *CDKN2A*. Overall, incubation of cells with 5 mM Nacr for 8 h effectively increased the expression of cell proliferation marker genes, thereby promoting cell proliferation.

### Effect of Nacr on cell cycle and related gene expression

**Figure 3:**
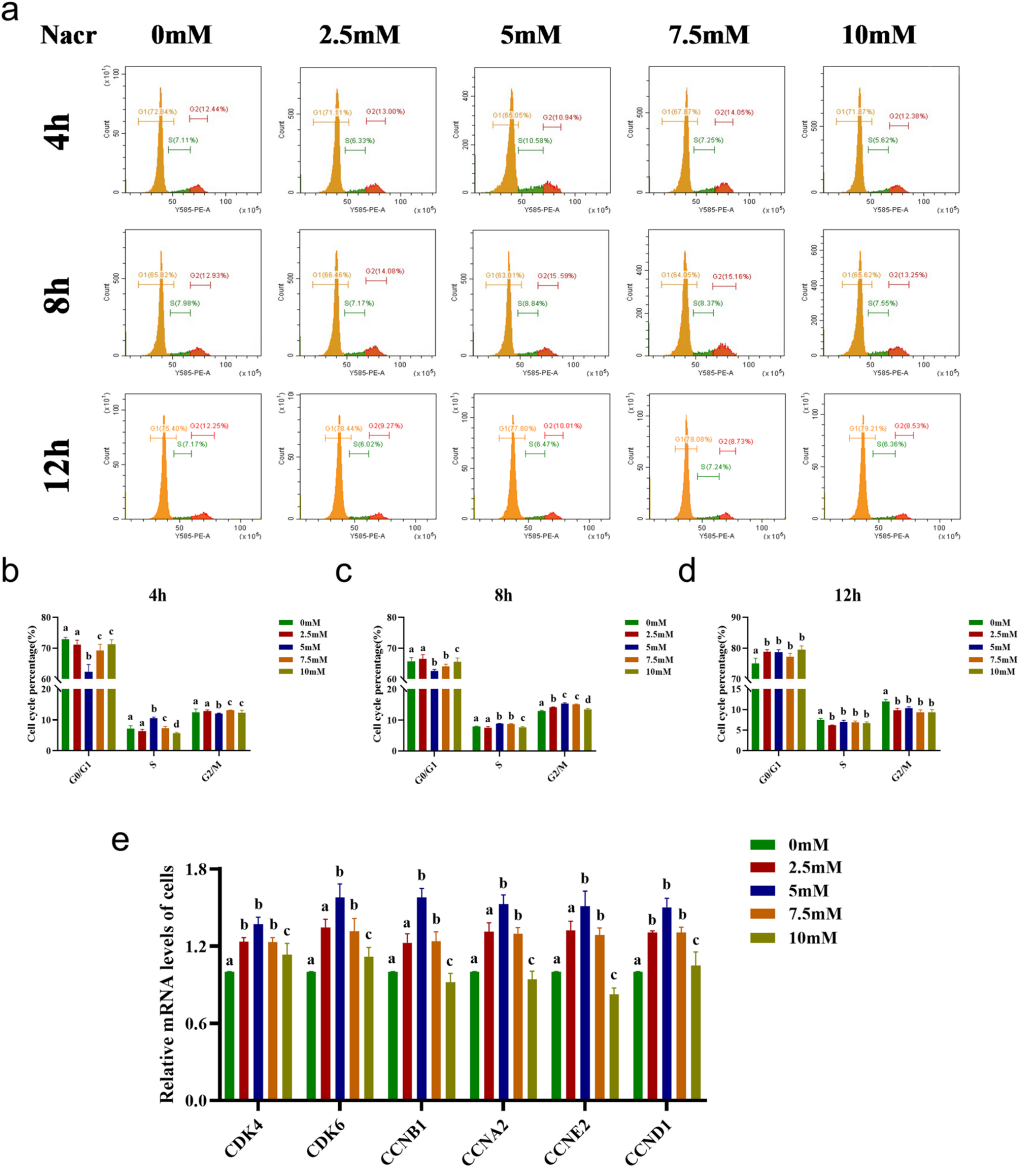
Effect of Nacr treatment on the cell cycle of bovine fibroblasts. (**a**) Cell cycle analysis detected by flow cytometry after treatment with different concentrations of Nacr for 4 h, 8 h and 12 h, respectively; (**b**) The proportion of cells in G0/G1, S, G2/M phases after 4 h treatment with different concentrations of Nacr; (**c**) The proportion of cells in G0/G1, S, G2/M phase of cells after 8 h treatment with different concentrations of Nacr; (**d**) The proportion of cells in G0/G1, S, G2/M phase of cells after 12 h treatment with different concentrations of Nacr; (**e**) Expression of cell cycle genes in bovine fibroblasts treated with different concentrations of Nacr for 8 h. Different letters represent significant differences.

Cell proliferation, which is intricately linked to the cell cycle, prompted an investigation into whether Nacr influences cell proliferation by modulating the cell cycle. Bovine fibroblasts cultured with 2.5 mM, 5 mM, 7.5 mM or 10 mM Nacr for 4 h, 8 h or 12 h were subjected to flow cytometry analysis to evaluate the cell cycle distribution. After 4 h of treatment, 5 mM Nacr significantly reduced the proportion of G0/G1 phase cells (65.05% vs. 72.84%, *P *< 0.05) and increased the proportion of S phase cells (10.58% vs. 7.11%, *P *< 0.05), while 10 mM Nacr decreased the proportion of S phase cells; other Nacr concentrations had no notable effect on the cell cycle. After 8 h of treatment, 5 mM and 7.5 mM Nacr increased the proportion of S-phase cells to 8.84% and 8.37% (7.98% and 7.89%), respectively, and the proportion of cells in the G2/M phase to 15.59% and 15.16% (12.93% and 12.93%), respectively. Additionally, the proportion of G0/G1 phase cells significantly decreased compared to that of the untreated fibroblasts. After 12 h of treatment, all Nacr concentrations increased the proportion of cells in the G0/G1 phase but decreased the proportion of cells in the S and G2/M phases, possibly due to the toxic effects that inhibited cell proliferation and blocked the G0/G1 phase (Figure 3a–d). Notably, treatment of bovine fibroblasts with 5 mM Nacr for 8 h resulted in a more favourable effect on the normal cell cycle, with significant increases in S and G2/M phase cells, thus promoting cell proliferation.

To validate these findings, we analysed the mRNA expression levels of cell cycle-related genes using RT‒qPCR. Compared to no treatment, 2.5 mM, 5 mM and 7.5 mM Nacr upregulated the mRNA expression of cyclin-dependent kinase 4 (*CDK4*), *CDK6*, cyclin B1 (*CCNB1*), cyclin A2 (*CCNA2*), cyclin E2 (*CCNE2*), and cyclin D1 (*CCND1*). However, 10 mM Nacr did not affect the expression of these cell cycle-related genes (Figure 3e). These results further support the conclusion that 8 h of treatment with different concentrations of Nacr has a positive effect on bovine fibroblast growth by modulating the cell cycle and influencing proliferation.

### Effect of Nacr on apoptosis-related gene expression in bovine fibroblasts

**Figure 4:**
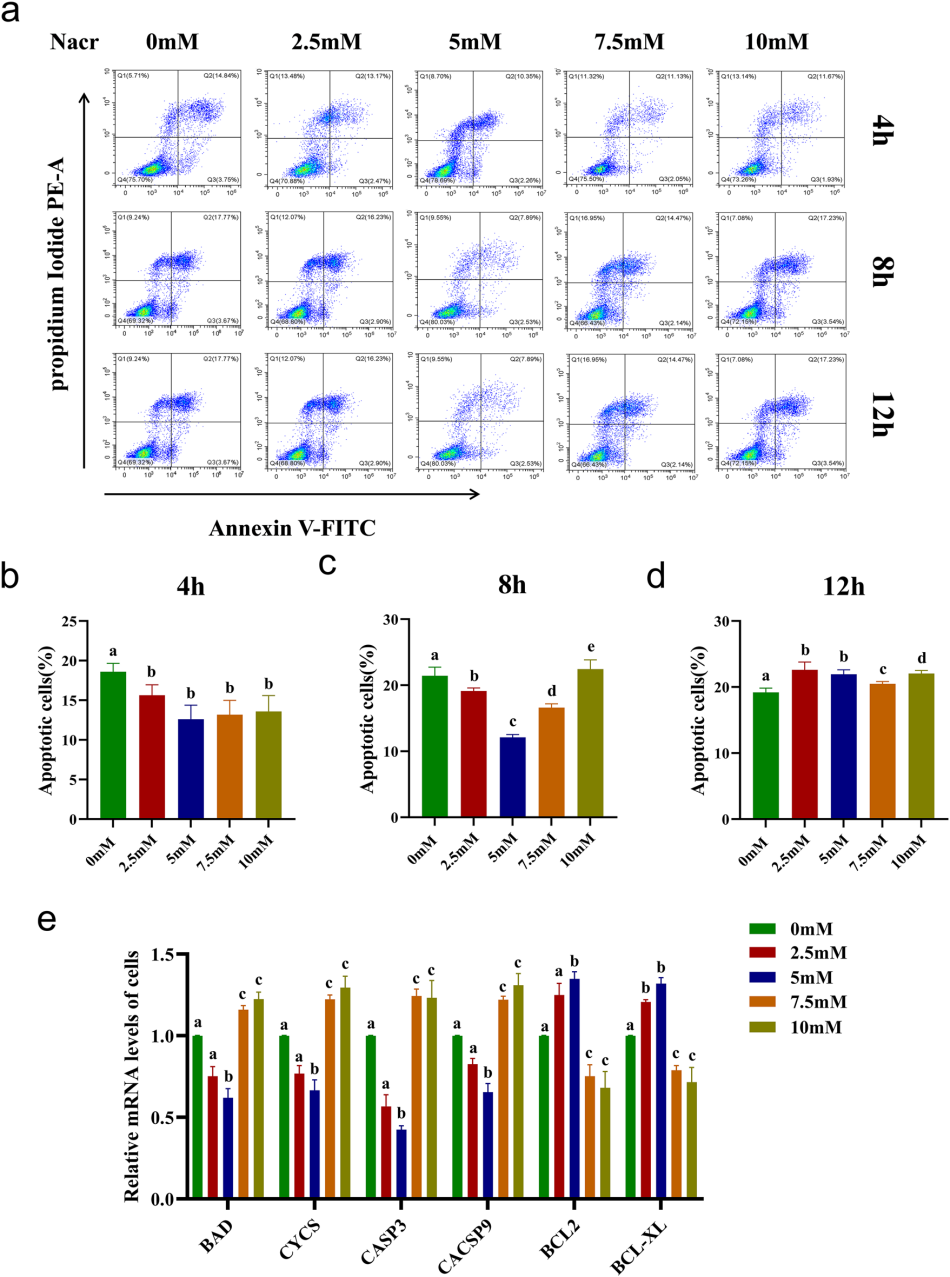
Effect of Nacr on apoptosis of bovine fibroblasts. (**a**) Apoptosis of bovine fibroblasts after treatment with different concentrations of Nacr for 4 h, 8 h and 12 h, respectively, in each quadrant Q1 is dead cells, Q2 is late apoptotic cells, Q3 is early apoptotic cells and Q4 is apoptotic is normal living cells, in this study Q2 and Q3 are recorded as total apoptotic cells; (**b**) Ratio total apoptotic cells after treatment with different concentrations of Nacr for 4 h, i.e. ration of early apoptotic cells (Q3) versus late apoptotic cells (Q2); (**c**) The ratio of total apoptotic cells after 8 h of Nacr treatment with different concentrations; (**d**) The ratio of total apoptotic cells after 12 h of Nacr treatment with different concentrations; (**e**) Apoptotic gene expression in bovine fibroblasts after 8 h Nacr treatment with different concentrations. Different letters represent significant differences.

The results described above indicate that Nacr may promote the growth and proliferation of bovine fibroblasts, but its effect on apoptosis is unclear. To investigate this further, we used Annexin V-FITC/PI double staining to assess the apoptosis of the cells treated with different concentrations of Nacr at different time intervals. After 4 h of treatment, compared to that of the cells without added Nacr, the percentage of total apoptotic cells significantly decreased from 18.6 to 12.61% in the 5 mM Nacr-treated group (*P *< 0.01). In the 7.5 mM Nacr-treated group, the percentage of apoptotic cells decreased from 18.6 to 13.18% (*P *< 0.01) and from 18.6 to 13.6% in the 10 mM Nacr-treated group (*P *< 0.05). After 8 h of treatment, the total percentage of apoptotic cells in the 5 mM Nacr-treated group was significantly lower than that in the untreated group (10.42% vs. 21.44%, *P *< 0.001). Early apoptosis decreased from 3.67 to 2.53%, while late apoptosis decreased from 17.77 to 7.89%. In the 7.5 mM Nacr-treated group, the total percentage of apoptotic cells was significantly reduced (13.18% vs. 21.44%, *P *< 0.01), the percentage of early apoptotic cells decreased to 2.05%, and the percentage of late apoptotic cells decreased to 11.13%. After 12 h of treatment, the percentage of apoptotic cells increased, with the highest percentages in the 2.5 mM, 5 mM and 7.5 mM Nacr-treated groups (Figure 4a–d). Continuous treatment of bovine fibroblasts with 5 mM Nacr for 8 h significantly reduced the percentage of apoptotic cells.

To explore the regulatory effect of Nacr on cell apoptosis, we used RT‒qPCR to analyse the expression of apoptosis-related genes after 8 h of treatment with 2.5 mM, 5 mM, 7.5 mM and 10 mM Nacr. Treatment with 2.5 mM and 5 mM Nacr downregulated the expression of the proapoptotic genes *BAD*, *CYCS*, *CASP3*, and *CASP9* while upregulating the expression of the apoptosis suppressor genes *BCL2* and *BCL-XL*. Conversely, 7.5 mM and 10 mM Nacr upregulated the expression of proapoptotic genes and downregulated the expression of apoptosis suppressor genes (Figure 4e). Continuous treatment with 5 mM Nacr for 8 h was particularly effective at reducing the expression of apoptosis-promoting genes, inhibiting cell apoptosis, and maintaining the normal growth of bovine fibroblasts.

### Effect of Nacr on histone crotonylation modification in bovine fibroblasts

**Figure 5:**
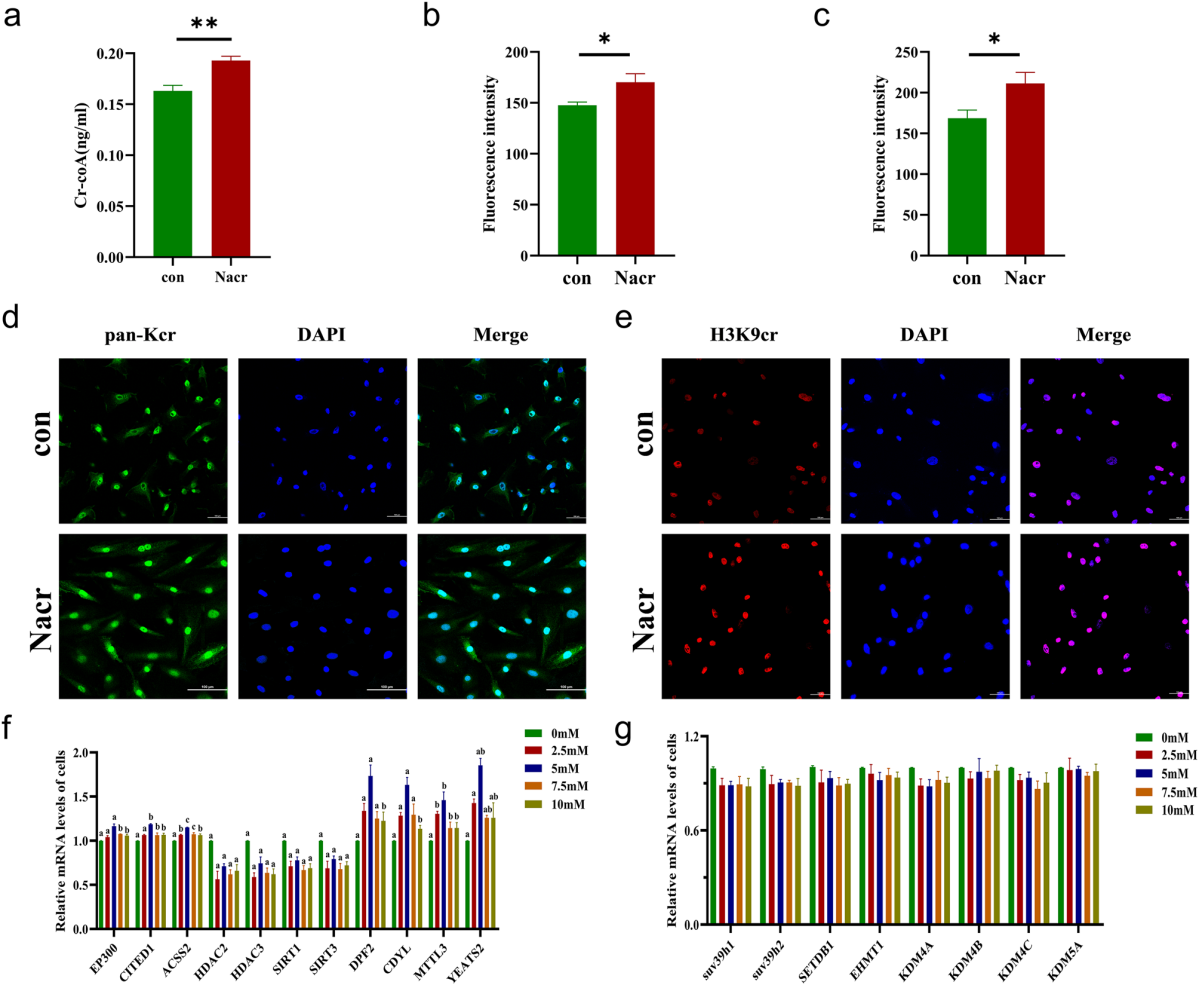
Nacr affect crotonyl modification in bovine fibroblasts. (**a**) ELISA detection of intracellular Cr-coA content after 8 h of 5 mM Nacr treatment; (**b**) Pan-Kcr fluorescence intensity Cr-coA after 8 h of 5 mM Nacr treatment; (**c**) H3K9cr fluorescent intensity after 8 h of 5 mM Nacr treatment; (**d**) Immunofluorescence detection of pan-Kcr modification level in the cells after 8 h of 5 mM Nacr treatment; (**e**) Immunofluorescence detection of H3K9cr modification level in cells after 5 mM Nacr treatment for 8 h; (**f**) Expression of Kcr modification related genes after different concentrations of Nacr treatment for 8 h; (**g**) Expression of histone methylation modifying enzymes after different concentrations of Nacr treatment for 8 h. Different letters represent significant differences.

In this study, we aimed to investigate whether Nacr regulates the overall Kcr modification level in cells and modulates cell proliferation via the Cr-CoA content. According to previous results, 5 mM Nacr treatment for 8 h is optimal for maintaining cell morphology, promoting cell proliferation, and inhibiting cell apoptosis. We examined the Cr-CoA content in the fibroblasts treated with 5 mM Nacr for 8 h. The results showed that the content of Cr-CoA in the cells significantly increased (Figure 5a). Furthermore, Nacr increased the overall modification level of Kcr in fibroblasts, especially significantly increasing H3K9cr modification (Figure 5b–e). To elucidate the mechanism by which Nacr regulates Kcr modification, we analysed the expression of enzymes related to Kcr modification. Nacr treatment at 2.5 mM, 5 mM, 7.5 mM and 10 mM upregulated the expression of Kcr-modified writer genes, including E1A binding protein p300 (*EP300*), Cbp/p300-interacting transactivator with Glu/Asp-rich carboxyterminal domain 1 (*CITED1*), and acyl-CoA synthetase 2 (*ACSS2*). Simultaneously, Nacr downregulated the expression of Kcr-modified eraser genes, such as histone deacetylase 2 (*HDAC2*), *HDAC3*, *SIRT1*, and *SIRT3*. Specifically, 2.5 mM and 5 mM Nacr upregulated the expression of Kcr-modified reader genes, including double PHD fingers 2 (*DPF2*), chromodomain Y-like protein (*CDYL*), MLLT3 super elongation complex subunit (*MLLT3*), and YEATS domain containing 2 (*YEATS2*) (Figure 5f).

In summary, 5 mM Nacr had the greatest effect on the regulation of Kcr-modified genes of writers, readers, and erasers. These results suggested that Nacr, a small molecule additive that regulates Kcr modification, could specifically modulate the level of Kcr modification in bovine fibroblasts by influencing the expression of Kcr-modified writer and eraser genes. To eliminate interference from other modifications, we also assessed enzymes associated with histone methylation, such as *SUV39H1* histone lysine methyltransferase, *SUV39H2*, SET domain bifurcated histone lysine methyltransferase 1 (*SETDB1*), euchromatic histone lysine methyltransferase 1 (*EHMT1*), lysine demethylase 4A (*KDM4A*), *KDM4B*, *KDM4C*, and *KDM5A*. The mRNA expression of these enzymes was reduced but not significantly different after treatment with different concentrations of Nacr for 8 h (Figure 5g). These results indicated that Nacr treatment of bovine fibroblasts specifically influenced histone crotonylation but did not affect the expression of histone methylation genes.

### Increased somatic cell nuclear transfer efficiency by the use of Nacr-treated cells

**Figure 6:**
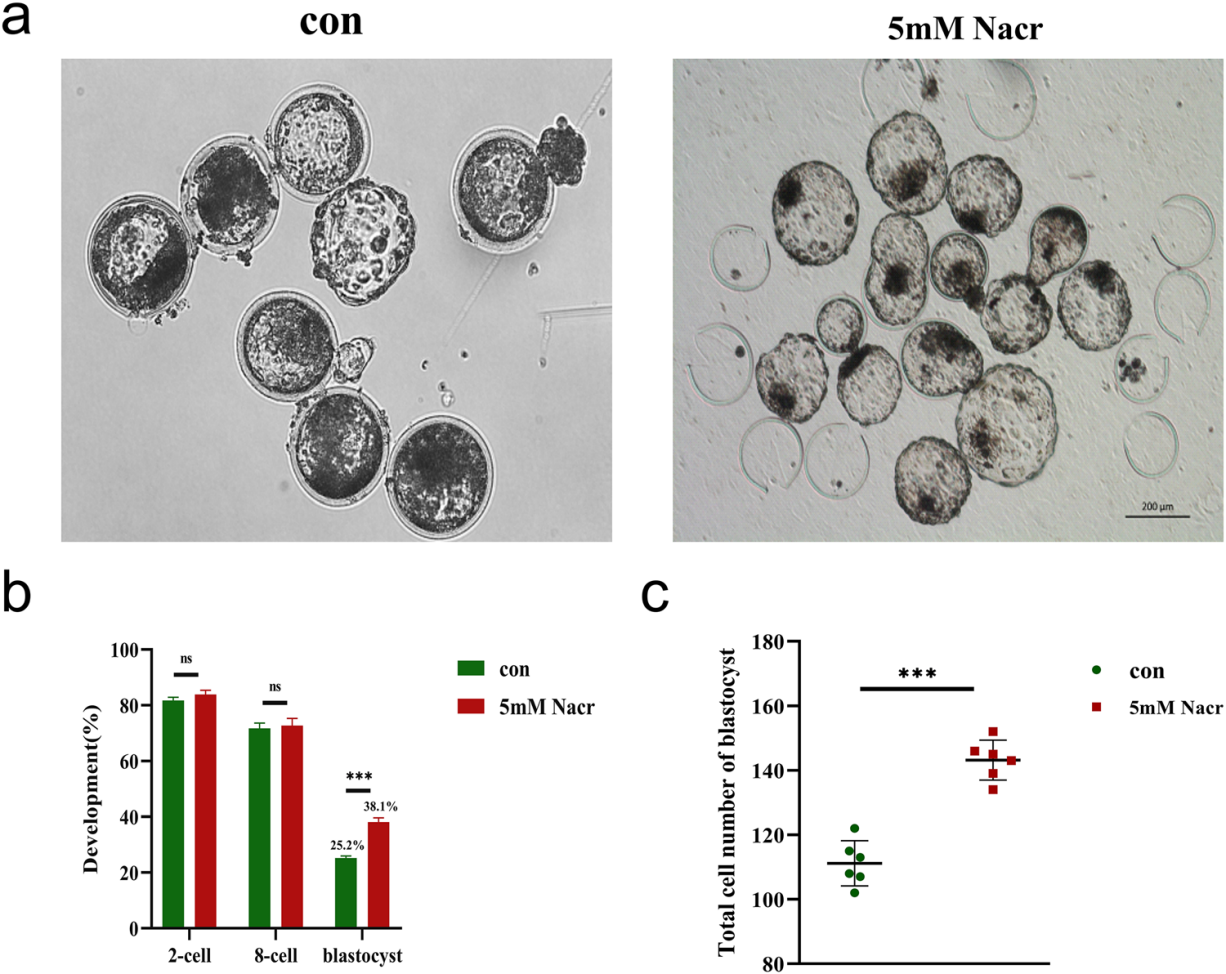
Nacr treatment of bovine fibroblasts improve SCNT embryo development. (**a**) Images of cloned blastocysts from Nacr-treated and control cells; (**b**) Proportions of 2-cell, 8-cell, and blastocyst stage embryos after SCNT with or without Nacr-treated cells; (**c**) Total cell number of SCNT blastocyst with or without Nacr-treated.

The aforementioned results demonstrated that the introduction of Nacr induces a notable increase in the level of Kcr in bovine fibroblasts, which is a pivotal factor that significantly increases cellular activity and cell proliferation in bovine fibroblasts. Subsequently, fibroblasts treated with 5 mM Nacr for 8 h were used as donor cells to produce cloned embryos via somatic cell nuclear transfer (SCNT) (Figure 6a). A total of 38.1% of the Nacr-treated embryos developed into blastocysts, and the total cell number of the SCNT blastocysts was significantly greater than that of the controls (25.2%) (Supplementary Table 1, Figure 6b-c). These findings confirmed that Nacr-treated fibroblasts significantly promoted SCNT embryo development, suggesting that Nacr induced increased Kcr modification and subsequently increased cellular developmental potential.

## Discussion

Kcr is a conserved chromatin marker that is ubiquitous on histones of various species and is predominantly found in transcriptionally active promoter regions and potential enhancer regions. This modification plays a pivotal role in the regulation of gene expression^35–38^. Progress has been made in identifying Kcr-modifying enzymes and modification sites and exploring their biological functions, but several questions remain unanswered. Specifically, the exact mechanisms by which Kcr modifications regulate cellular metabolism and cell proliferation remain unclear. Wei et al.^27^ and Liu et al.^12^ suggested that the exogenous addition of Nacr inhibited the proliferation of cancer cells and increased the viability of sperm sex chromosomes. In addition, Wen et al. reported that intraperitoneal injection of Nacr in mice with myocardial I/RF injury significantly inhibited apoptosis and promoted cell proliferation after 7 and 14 days^39^. In the present study, the addition of exogenous Nacr during the culture of bovine ear tip fibroblasts significantly promoted cell growth and promoted cell proliferation. Notably, the most effective treatment was 5 mM Nacr for 8 hours, which increased the proportion of S-phase and G2/M-phase cells and facilitated the smooth transition from G1 to S-phase and to G2/M-phase. These changes were accompanied by a reduction in apoptosis and no signs of growth retardation.

Cell proliferation and apoptosis are strictly regulated by gene expression, involving cyclins, caspase proteases and the Bcl-2 family^40–42^. Metabolic pathways provide biomolecules necessary for cell proliferation and division. When the nutrient composition changes, cells can adjust their metabolic status to ensure normal growth, partly due to the epitope modification of histone proteins^43^. Our examination of mRNA expression levels revealed that exogenous Nacr upregulated the expression of proliferation marker genes, such as *PCNA*, *CDK1*, *MK167* and *CDK2*, while downregulating proliferation inhibitor genes, such as *CDKN1A* and *CDKN2A*. The expression of antiapoptotic genes (*BCL-2* and *BCL-XL*) tended to increase, and the expression of apoptosis-promoting genes (*BAD*, *CYCS*, *CASP3*, and *CASP9*) decreased when Nacr was used. In contrast, cycle regulation-related genes (*CDK4*, *CDK6*, *CCNB1*, *CCNA2*, *CCNE2*, and *CCND1*) were significantly upregulated, indicating that the addition of Nacr can regulate cell proliferation by affecting the expression of apoptosis- and cell cycle-related genes, which might be due to the toxicity of Nacr to cells.

Kcr modification involves the addition of a crotonyl group to lysine residues, which is facilitated by crotonyltransferase (HTC) using crotonyl-coenzyme A (Cr-CoA) as a substrate. Cr-CoA serves as a cellular intermediary metabolite in various metabolic pathways^44^. Nacr, a kind of crotonate, acts similarly to de-Kac modification inhibitors such as sodium butyrate (NABU) and trichostatin A (TSA), increasing Kcr modification levels^45^. This study, akin to findings by Tina et al., revealed that Nacr treatment significantly increased the Cr-CoA content in bovine fibroblasts, resulting in a substantial increase in intracellular Kcr modification levels^46^. This change, in turn, correlated with increased cell proliferation and promoted cell growth. The regulation of Kcr modification is influenced not only by the intracellular Cr-CoA concentration but also by the expression of writers, erasers, and readers^47^. Our results indicated that Nacr upregulated the expression of Kcr-modified writers (*EP300*, *CBP*, and *ACSS2*) and reader genes (*DPF2*, *CDYL*, *MLLT3* and *TEATS2*) and downregulated the expression of eraser genes (*HDAC2*, *HDAC3*, *SIRT1* and *SIRT3*). These results suggested that Nacr modulates the cellular Kcr modification level by influencing the expression of Kcr modification-related enzymes. Despite a previous report demonstrating the negative regulatory role of the *CDYL* gene in Kcr modification^15^, our results demonstrated increased expression of the *CDYL* gene and Kcr modification following the addition of Nacr. These changes may be influenced by the Nacr concentration, *CDYL* gene mechanisms and other factors.

The low success rate of SCNT poses a major challenge to the widespread application of this biological breeding technique. Our study revealed that the addition of Nacr increased the viability and proliferative capacity of bovine fibroblasts, leading to a substantial increase in SCNT blastocyst development. This improvement may be attributed to the heightened cellular Cr-CoA content and elevated Kcr modification levels induced by Nacr. Our results showed that the addition of Nacr during the SCNT donor fibroblast culture period significantly increased the efficacy of SCNT embryo development. However, whether this treatment can affect the success rate of postimplantation SCNT pregnancies as well as foetal birth or survival rates needs to be further explored in depth.

In conclusion, our findings indicate that exogenous Nacr can increase the Kcr modification level in bovine fibroblasts by increasing the Cr-CoA content and modulating the expression of Kcr-modifying enzymes. The use of Nacr-treated fibroblasts as donor cells significantly promoted the development of clonal blastocysts, providing a new approach for exploring the regulatory role of Kcr and promoting the developmental efficacy of SCNT embryos before transplantation.

## Methods

### Bovine fibroblasts culture

The fibroblasts selected for this study were obtained from bovine ear tips. Bovine ear tip fibroblast progenitor cells were preisolated and cultured in our laboratory. All animal experiments were performed according to the National Institutes of Health’s Guide for Care and Use of Laboratory Animals and the Animal Research: Reporting in Vivo Experiments (ARRIVE) Guidelines, and procedures were approved by the Institutional Animal Care and Inner Mongolia University (Approval: IMU-CATTLE-2022-057).

Bovine ear tip tissues were obtained from Chinese Simmental and crossbred cattle bred at the ranch of Chifeng Shengquan Ecological Ranching Co. in Chifeng City, Inner Mongolia Autonomous Region, China (42° 260-43° 250). The ear tip tissues were removed from the fur, washed with PBS supplemented with 2% penicillin/streptomycin (Gibco, 15140122), cut into 1 mm^2^ pieces with scissors, placed at the bottom of a Petri dish, inverted and placed in an incubator at 37.5 °C and 5% CO_2_. After the liquid around the tissue was allowed to dry, the culture solution was added and incubated at 37.5 °C in a 5% CO_2_ incubator. The cell density reached approximately 90% for passaging and freezing. Bovine fibroblasts were cultured in DMEM-F12 (Gibco, A4192001) supplemented with 10% foetal bovine serum (Opcel Biotechnology Co., Ltd., Hohhot, China, 1101) and 1% penicillin/streptomycin. Cells were passaged at a ratio of 1:3 when the cell density reached 90%.

### Cell density statistics

Using a ZEISS (Axio observer Z1) inverted fluorescence microscope (Gemany), images of cells were captured at 4x magnification after culturing with 0 mM, 2.5 mM, 5 mM, 7.5 mM, and 10 mM Nacr for 4 hours, 8 hours, and 12 hours, respectively. The cell counts in each field of view were quantified using ImageJ software. Three fields of view were selected for each group, and the process was repeated three times.

### Live cell count

After trypsin digestion, the cells were collected from cultures incubated with 0 mM, 2.5 mM, 5 mM, 7.5 mM or 10 mM Nacr for 4 h, 8 h or 12 h. Then, 10 µl of 0.4% Trypan blue stain (Thermo Fisher Scientific, T10282) was added to the resuspended cells (10 µl), and the mixture was thoroughly mixed before adding 10 µl of the cell suspension to the cell counting plate. The percentage of dead cells was determined using a cell counter (Life Technologies Countess II, USA).

### Edu detects cell proliferation

For the EdU assays, Nacr cells were treated with 2.5 mM, 5 mM, 7.5 mM, or 10 mM for 4 h, 8 h, or 12 h, and cell proliferation was assessed using the APE BIO EdU Imaging Kit (Cy3) (Apexbio K1975) according to the manufacturer’s protocol.

### Detection of cell cycle and apoptosis by flow cytometry

When the confluence of bovine fibroblasts reached 70–80%, different concentrations of Nacr were added to the cells for 4 h, 8 h and 12 h, and the cell cycle distribution and apoptosis were assessed using a Cell Cycle and Apoptosis Kit (Beyotime, C1052) and an Annexin v-FITC Apoptosis Kit (Beyotime, C1062D), respectively.

### Immunofluorescence of cells

The cells were fixed with 4% paraformaldehyde for 10 minutes and then permeabilized with 0.1% Triton X-100 in PBS for 10 minutes after washing. The cells were then incubated with 5% BSA in PBS at room temperature for 1 h, followed by incubation with rabbit anti-pan-Kcr antibody (PTM BIO, PTM-502, diluted 1:500) and rabbit anti-H3K9cr antibody (PTM BIO, PTM-539, diluted 1:200) overnight at 4 ℃. After washing, the cells were incubated with an Alexa Fluor 488-conjugated secondary antibody (Proteintech, SA00013-2, diluted 1:1000) and an Alexa Fluor 549-conjugated secondary antibody (Proteintech, SA00013-4, diluted 1:1000) at room temperature for 1 h in the dark. The cells were placed on glass slides and then examined with a confocal laser scanning microscope (Nikon A1 plus, Japan).

### Cr-coA levels evaluation

Intracellular Cr-CoA levels were measured by using a bovine Cr-CoA kit (MM-5108901, MEIMIAN) according to the manufacturer’s instructions.

### Quantitative RT-PCR

Total RNA was extracted from cultured bovine fibroblasts using RNAiso Plus (Vazyme) according to the manufacturer’s instructions, and a Nanodrop (Thermo, Germany) was used to quantify the purity and concentration of the RNA. One microgram of cDNA from each sample was obtained using HiScript® II Q RT SuperMix for qPCR (+gDNA wiper) (Vazyme). Real-time PCR was performed using ChamQ UnIversal SYBR qPCR Master Mix (Vazyme) from Roche (LghtCycler 480) (USA) following the manufacturer’s instructions, and the relative expression of the target gene was subsequently normalized to that of the housekeeping gene (Gapdh) using the 2 ^−△△C^ method. The primers used for qPCR are listed in Supplementary Table 2.

### Oocytes acquisition and in vitro maturation

The ovaries used in this study were obtained from abattoirs. The bovine cumulus-oocyte complexes (COCs) were aspirated from follicles 3–8 mm in diameter in the ovary using a 1.2 mm syringe. Oocytes were placed in IVM (M199+10 mmol/l HEPES+10% FBS+0.01 µg/mL FSH+1 µ/mL LH+ 0.01 µg/mL E2) culture medium at 38.5 ℃ and 5% CO_2_ for 20–22 h for in vitro maturation.

### SCNT and embryo culture

The SCNT method was performed as previously described^48^. Nacr-treated bovine ear tip fibroblasts were digested into single cells, and the individual cells were transferred into the zona pellucida interspaces of enucleated oocytes. The successfully reconstituted embryos were then electrofused and cultured in SOF developmental solution for 30 minutes at 38.5 ℃ with 5% CO_2_ under saturated humidity. The fused embryos were activated in 5 µmol/L ionomycin for 5 min, followed by incubation in 10 µg/mL actinomycin for 5 h. Subsequently, at 38.5 ℃ with 5% CO_2_ under saturated humidity, the embryos were transferred to SOF developmental medium supplemented with mineral oil for further culture until development to the blastocyst stage. The number of embryos at each stage was counted.

### Statistics and reproducibility

All values are expressed as the mean ± SEM. GraphPad Prism 8 software was used to analyse the significance of differences (one-way ANOVA was used for multiple group comparisons, and Student's t test was used for two-group comparisons), and *P *< 0.05 was considered to indicate a significant difference (*P < 0.05, **P < 0.01, ***P < 0.001). The experiment was repeated three times for both technical and biological replicates.

### Supplementary Information

Supplementary Information.

## Data Availability

All data generated or analyzed during this study are included in this published article and its supplementary information files.
