# Supplementary material for: High histone crotonylation modification in bovine fibroblasts promotes cell proliferation and the developmental efficiency of preimplantation nuclear transfer embryos
Source: Sci Rep. 2024 May 4;14:10295. doi: 10.1038/s41598-024-61148-6 (PMC11069573; doi:10.1038/s41598-024-61148-6)
Supplement: Supplementary file 1 — Supplementary Information. [file 41598_2024_61148_MOESM1_ESM.docx]

Supplementary Table 1: Development of SCNT embryos from different donor cell

| **Items** | **Donor cells** | |
| --- | --- | --- |
|  | **con Bovine fibroblasts** | **5mM Nacr Bovine fibroblasts** |
| The number of replicates | 3 | 3 |
| Number of fused clones | 477^a^ | 511^a^ |
| 2-cell rate (%) | 81.7±0.94^a^ | 83.8±1.29^a^ |
| 8-cell rate (%) | 71.7±1.55^a^ | 72.7±2.08^a^ |
| Blastocyst rate (%) | 25.2±0.62^a^ | 38.1±1.25^b^ |

Note: Different letters indicate significant difference, ^a, b^ in the same column (P<0.05).

| GENE NAME | Forward primer 5'-3 | Reverse primer 5'-3' |
| --- | --- | --- |
| GAPDH | GATGGTGAAGGTCGGAGTGAAC | GTCATTGATGGCGACGATGT |
| PCNA | AGTCACATTGGAGATGCTGTTGTA | AGCTGAACTGGTTCATTCATCTCTA |
| MK167 | CCAGGCTGCTGTAAATGCTTC | CGGACACCCTGTCATTGTTC |
| CDKN2A | CGTGCTGATGGCTAGTGAGGA | CCATCATCATCACCTGGTCTAGGA |
| CDKN1A | TTGCACTTTGAATAACAGCTGAAGG | CAAGTGGTTCAGCGCAAGGA |
| CDK2 | TGGGTCCATCAAGCTAGCAGA | TGGAGTAGTATTTGCAGCCCAGA |
| BCL2 | CTGTGGATGACCGAGTACCTGAAC | AGACTGAGCAGTGCCTTCAGAGAC |
| BCL-XL | CATCACAGGGTTGGGCCTAGA | TGATGCCACGACAATTGAGGA |
| CYCS | GGTTGCACCAACACCGGTA | CACTGGGCACACTTCTGAACA |
| CASP3 | CCAGGGTGCCCAGGACTTTA | GTGTCCCATCTCTGGCCTTCA |
| CASP9 | CCCAGGGACTTCTGGTGGTTAG | TCTGCACACAAGGCTCTGCTC |
| BAD | TCCACACAACGGTGACCTTC | TGCGTCTTCACAGTCTGCTTC |
| CDK4 | GAGCTGGAGGAATCTGGAGCAC | AGGGCTCGGAAGGCAGAGAT |
| CDK6 | GAACACGTTGATCAAGACTTGACCA | GAAAGTCCAGGCCTCGGAGA |
| CCNB1 | GCGGATCCAAACCTTTGTAGTG | AATGAGGATGGCTCTCATGTTTC |
| CCNE2 | GCACAGCACCTCATGGAACTTAAC | TGGCAGTGAAACTTGGGAATTG |
| CCND1 | CTGTGCATTTACACTGACAACTCCA | GTTCACCAGGACCAGCTCCA |
| CCNA2 | GACCCTGCATTTGGCTGTGA | CTCTGCTACTTCTGGCGGGTATATC |
| EP300 | AGAACTGTGCAGTAGCTGTTTGTGG | GGAGCATGTGGTTGGCTTCA |
| CBP | GCCAAGACTCTGAAATGCCAACTA | CCTTGGTTCCATTCGAGGCTAC |
| ACSS2 | ACGAACGCTTTGAGACCACCTAC | CAACAGCCTTGTGTTCCACGA |
| HDAC2 | AGCCCATGGCGTACAGTCAA | TGGGATGACCCTGTCCGTAATAA |
| HDAC3 | CTTCAACCTCAGCATTCGAGGAC | GTGTAGCCACCACCACCTAGCA |
| SIRT1 | CACTGGAGCAGGTTGCAGGA | TTCATCAGCTGGGCATCTAGGAC |
| SIRT3 | CTGGACAGACGACATCCAGGAC | TCCTGGTTCAGCCACAATTTGTAA |
| DPF2 | CCTGTGACGAACAGTCGAGCA | CTCATCATCAAGGTCATCCAGGAA |
| MLLT3 | CGATGAACTGGTAGAGCTTCACAGA | GTCCAGCGAGCAAAGGTCA |
| YEATS2 | GGCCCAGTCACCAAAGATTCA | GATTGCCACAGCCTGCTTCA |
| CDYL | ACCTTCGGGCAGAGTCCAGA | GTTAGCTTTCGCCCGCTGAG |
| SUV39H1 | CCATCCATGGGTTGCACTTAC | CCATGAATCCCAACTGCAGA |
| SUV39H2 | GATGCAGCTCGATATGGAAATGTG | GACGGGTATCGAGGTTATCAATGAA |
| EHMT1 | ATTTCTGACTCGGAAGCTGATGTG | TGGTTGATGAAGCGGCTGAC |
| SETDB1 | CCGTGGACAGTGATGATATCCAG | ATGCCATGGGTTGATTTCAAAG |
| KDM4A | TTCCGGCACAAGATCCCTAA | GCCTTACCACCTCACACTGGTAGA |
| KDM4B | AAACATGTTGGTCATTCCAGGCTA | TCTCTGGCCTGGCAACTGTTA |
| KDM5A | CGGAGGAGTCGGATGATGAGA | TTTCCGCCATTTCTGGAGAGAC |
| KDM4C | GGATCAAATGTTGCCCACATGTA | CGCATGTCAGAAGCTGTGGA |

Supplementary Table 2: List of RT-qPCR specific primers used in this study
